# Genetic alterations of m^6^A regulators predict poorer survival in acute myeloid leukemia

**DOI:** 10.1186/s13045-017-0410-6

**Published:** 2017-02-02

**Authors:** Chau-To Kwok, Amy D. Marshall, John E. J. Rasko, Justin J. L. Wong

**Affiliations:** 1 Gene & Stem Cell Therapy Program, Centenary Institute, University of Sydney, Camperdown, 2050 Australia; 2 Gene Regulation in Cancer Laboratory, Centenary Institute, University of Sydney, Camperdown, 2050 Australia; 3 Sydney Medical School, University of Sydney, Camperdown, NSW 2006 Australia; 4 Cell and Molecular Therapies, Royal Prince Alfred Hospital, Camperdown, 2050 Australia

**Keywords:** RNA modification, m^6^A, Leukemia, Acute myeloid leukemia, TP53 mutation

## Abstract

Methylation of N^6^ adenosine (m^6^A) is known to be important for diverse biological processes including gene expression control, translation of protein, and messenger RNA (mRNA) splicing. However, its role in the development of human cancers is poorly understood. By analyzing datasets from the Cancer Genome Atlas Research Network (TCGA) acute myeloid leukemia (AML) study, we discover that mutations and/or copy number variations of m^6^A regulatory genes are strongly associated with the presence of TP53 mutations in AML patients. Further, our analyses reveal that alterations in m^6^A regulatory genes confer a worse survival in AML. Our work indicates that genetic alterations of m^6^A regulatory genes may cooperate with TP53 and/or its regulator/downstream targets in the pathogenesis and/or maintenance of AML.

**Electronic supplementary material:**

The online version of this article (doi:10.1186/s13045-017-0410-6) contains supplementary material, which is available to authorized users.

## To the editor

Methylation of N^6^ adenosine (m^6^A) is the most abundant form of messenger RNA (mRNA) modification in eukaryotes [1]. It is known to play crucial roles in the regulation of gene expression, protein translation, and splicing in normal biology [1, 2]. m^6^A regulatory enzymes consist of “writers” METTL3 and METTL14, “readers” YTHDF1 and YTHDF2, and “erasers” FTO and ALKBH5 [1]. m^6^A perturbation mediated via knockdown or knockout of these enzymes can cause cell death, decreased cell proliferation, impaired self-renewal capacity, and developmental defects [1]. For example, ablation of METTL3 perturbs embryonic stem cell differentiation [1]. Depletion of FTO and ALKBH5 leads to obesity and impairment of spermatogenesis, respectively [1]. Silencing of m^6^A methyltransferase can result in modulation of the TP53 signaling pathway of relevance to tumorigenesis [2]. More recently, overexpression of FTO has been shown to promote leukemogenesis [3]. It is therefore surprising that genetic alterations affecting m^6^A regulatory genes have not been explored in human cancers, including leukemia. Hence, there is a compelling reason to determine whether mutations, deletions, and amplifications of m^6^A regulatory genes are enriched in leukemia subtypes. Clinicopathological associations including patient survival have not previously been reported.

Here, we curate mutations, including point mutations, deep deletions, and amplifications of the best characterized m^6^A regulatory genes, *METTL3*, *METTL14*, *YTHDF1*, *YTHDF2*, *FTO*, and *ALKBH5*. Deep deletions are possibly homozygous deletions as measured using the Genomic Identification of Significant Targets in Cancer algorithm (GISTIC). Four distinct types of hematological malignancies were sequenced by the Cancer Genome Atlas Research (TCGA) Network: acute myeloid leukemia (AML), multiple myeloma (MM), acute lymphoblastic leukemia (ALL), and chronic lymphocytic leukemia (CLL), and genetic data has been made available via cBioPortal [4]. Mutations of m^6^A regulatory genes were found in 2.6% (5/191) of AML, 2.4% (5/205) of MM, 1.0% (1/106) of ALL, and 0% (0/666) of CLL (Additional file 1: Figure S1a). For AML, we further identified variation in gene copy number in 10.5% (20/191) of patients (Additional file 2: Table S1). There was a comparable frequency of copy number loss measured as shallow deletion (possibly heterozygous deletion) using GISTIC (*n* = 19) and copy number gain (*n* = 13) of m^6^A regulatory genes (Additional file 1: Figure S1b). Among these, copy number loss of *ALKBH5* is the most frequent in this AML cohort (12/191, 6.3%). Notably, 4.7% (9/191) of AML patients had concomitant copy number gain or loss of more than one m^6^A regulatory gene (Additional file 2: Table S1). In four of these nine cases, a copy number gain of an m^6^A writer was detected concomitantly with a shallow/deep deletion of an m^6^A eraser (Additional file 2: Table S1), indicating a potential synergistic alteration of m^6^A regulatory enzymes that may lead to increased levels of RNA m^6^A modification. Shallow deletions of *METTL14*, *FTO*, and *ALLBH5* were significantly associated with reduced mRNA expression of these genes (Additional file 3: Figure S2). Copy number gain of *METTL14* was significantly associated with an increase in its expression (Additional file 3: Figure S2). Thus, shallow deletion and copy number gain may result in the reduced and increased expression of m^6^A regulatory genes, respectively.

We determined whether mutations and copy number variations (CNVs) of m^6^A regulatory genes are associated with clinicopathological and molecular features of AML. Mutations and/or CNVs of *METTL3*, *METTL14*, *YTHDF1*, *YTHDF2*, *FTO*, and *ALKBH5* as a group were significantly associated with poorer cytogenetic risk in AML (*P* < 0.0001, Table 1). Additionally, we observed a marked increased in *TP53* mutations (*P* < 0.0001, Table 1) but a significant lack of *NPM1* and *FLT3* mutations (*P* < 0.005, Table 1) in AML patients harboring genetic alterations of m^6^A regulatory genes. These clinicopathological and molecular features were also associated with CNVs of m^6^A regulatory genes alone (Table 1). However, they were not associated with mutations of m^6^A regulatory genes alone (Table 1), which may be due to the small number of cases with mutations (*n* = 5).

**Table 1:** Clinical and molecular characteristics of TCGA AML patients according to the mutation and/or copy number variation status of genes encoding m^6^A regulatory enzymes

	Mutation and/or CNV				CNV only^a^			Mutation	
	Yes (*n* = 23)	No (*n* = 168)	*P*	Yes (*n* = 18)	No (*n* = 168)	*P*	Yes (*n* = 5)	No (*n* = 186)	*P*
Age			0.083			0.193			0.205
Median (range)	65 (18–81)	57 (21–88)		62.5 (18–81)	57 (21–88)		65 (45–76)	57.5 (18–88)	
Sex, no. (%)			0.123			0.321			0.376
Male	16 (8.4)	87 (45.5)		12 (6.5)	87 (46.8)		4 (2.1)	99 (51.8)	
Female	7 (3.7)	81 (42.4)		6 (3.2)	81 (43.5)		1 (0.5)	87 (45.5)	
BM blast			0.072			**0.038**			0.915
Median % (range)	60 (30–97)	73 (30–100)		54 (30–97)	73 (30–100)		75 (33–90)	72 (30–100)	
WBC, ×10^3^/mm^3^			0.084			**0.047**			0.889
Median (range)	5.4 (0.7–202.7)	17.5 (0.4–298.4)		5.2 (2.3–101.3)	17.45(0.4–298.4)		14.5 (2.3–101.3)	15.6 (0.4–298.4)	
Cytogenetic risk, no. (%)			**<0.0001**			**<0.0001**			0.483
Favorable	0 (0)	37 (19.4)		0 (0)	37 (19.9)		0 (0)	37 (19.4)	
Intermediate	4 (2.1)	105 (55)		1 (0.5)	105 (56.5)		3 (1.6)	106 (55.5)	
Unfavorable	19 (9.9)	21 (11)		17 (9.1)	21 (11.3)		2 (1)	38 (19.9)	
Missing data	0 (0)	5 (2.6)		0 (0)	5 (2.6)		0 (0)	5 (2.6)	
Mutation, no./total no. (%)									
*FLT3*	1/23 (4.3)	53/168 (31.5)	**0.005**	0/18 (0)	53/168 (31.5)	**0.002**	1/5 (20)	53/186 (28.4)	1.000
*NPM1*	1/23 (4.3)	51/168 (30)	**0.006**	0/18 (0)	51/168 (30.3)	**0.004**	1/5 (20)	51/186 (27.4)	1.000
*DNMT3A*	4/23 (17.4)	43/168 (25.6)	0.453	2/18 (11.1)	43/168 (25.6)	0.249	2/5 (40)	45/186 (24.2)	0.598
*IDH1* or *IDH2*	1/23 (4.3)	34/168 (20.2)	0.084	0/18 (0)	34/168 (20.2)	**0.048**	1/5 (20)	34/186 (18.3)	1.000
*NRAS* or *KRAS*	3/23 (13)	20/168 (11.9)	0.744	3/18 (16.7)	20/168 (11.9)	0.471	0/5 (0)	23/186 (12.4)	1.000
*RUNX1*	2/23 (8.7)	17/168 (10.1)	1.000	0/18 (0)	17/168 (10.1)	0.380	2/5 (40)	17/186 (9.1)	0.078
*TET2*	1/23 (4.3)	15/168 (8.9)	0.698	1/18(5.6)	15/168 (8.9)	1.000	0/5 (0)	16/186 (8.6)	1.000
*TP53*	15/23 (65.2)	1/168 (0.6)	**<0.0001**	13/18 (72.2)	1/168 (0.6)	**<0.0001**	2/5 (40)	14/186 (7.5)	0.057
*CEBPA*	2/23 (8.7)	10/168 (6.0)	0.641	2/18 (11.1)	10/168 (6.0)	0.327	0/5 (0)	12/186 (6.5)	1.000
*WT1*	0/23 (0)	12/168 (7.1)	0.366	0/18 (0)	12/168 (7.1)	0.610	0/5 (0)	12/186 (6.5)	1.000
*PTPN11*	2/23 (8.7)	6/168 (3.6)	0.248	2/18 (11.1)	6/168 (3.6)	0.175	0/5 (0)	8/186 (4.3)	1.000
*KIT*	1/23 (4.3)	6/168 (3.6)	0.599	0/18 (20)	6/168 (3.6)	1.000	1/5 (20)	6/186 (3.2)	0.172

Significant *P* values are in bold

*CNV* copy number variation, *BM* bone marrow, *WBC* white blood cell

^a^Excluding samples with m^6^A regulatory gene mutations

We further determined whether shallow/deep deletion of *ALKBH5* is associated with the clinicopathological and molecular features. Consistent with our findings in m^6^A regulatory genes overall, shallow/deep deletion of *ALKBH5* was significantly associated with poorer cytogenetic risk and the presence of *TP53* mutation in this AML cohort (*P* < 0.0001, Additional file 4: Table S2). *NPM1* and *FLT3* mutations were absent in AML patients with shallow/deep deletion of *ALKBH5* (Additional file 4: Table S2).

**Fig. 1:**
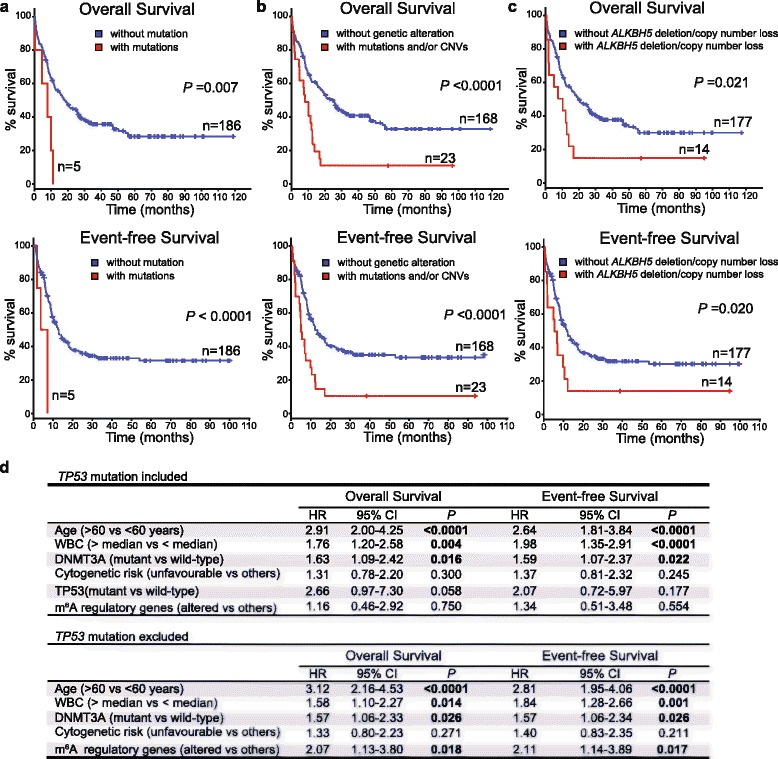
Kaplan-Meier curves for overall and event-free survival of TCGA AML patients by the presence and absence of **a** mutation of m^6^A regulatory genes, **b** mutation and/or copy number variation (CNV) of m^6^A regulatory genes, and **c** deletion/copy number loss of the *ALKBH5* gene encoding an important m^6^A “eraser.” Mutations include point mutation, deep deletion, and amplification. Log-rank test was used to determine significance. +, censored data. **d** Multivariate analysis for overall and event-free survival in TCGA AML patients

We performed Kaplan-Meier analysis to investigate the impact of genetic alterations in m^6^A regulatory genes on overall (OS) and event-free survival (EFS) in patients with AML. As a group, patients with a mutation of any of the genes encoding m^6^A regulatory enzymes had a worse OS (*P* = 0.007) and EFS (*P* < 0.0001, Fig. 1a). Inferior OS and EFS were also evident in patients who had mutations and/or CNVs of these genes (Fig. 1b) and in those with shallow/deep deletion of *ALKBH5* (Fig. 1c).

Of all clinicopathological and molecular features considered for this de novo AML cohort [5], older age (>60 years), white blood cell count > median (15,200 per mm^3^), unfavorable cytogenetic risk, and *DNMT3A* and *TP53* mutations were significantly associated with inferior OS and/or EFS in univariate analyses (Additional file 5: Figure S3 and Additional file 6: Figure S4). We therefore examined the impact of m^6^A regulatory gene mutations and/or CNVs on the outcome of AML patients with poor risk genotypes. Alterations of m^6^A regulatory genes as a group were associated with inferior OS and EFS in patients regardless of age (Additional file 7: Figure S5). These genetic alterations did not confer a worse OS or EFS in patients with unfavorable cytogenetic risk, white blood cell count > median, or *DNMT3A* mutations (Additional file 8: Figure S6).

We further determined the survival of AML patients based on whether they exhibited combined *TP53* mutations and genetic alterations of m^6^A regulatory genes. Almost all patients with mutated *TP53* (93.6%, Table 1) had ≥1 genetic alteration(s) of m^6^A regulatory gene(s). This group of patients had worse OS and EFS than patients who did not have any of these genetic alterations (Additional file 9: Figure S7a). There is a non-significant trend in patients with wild-type *TP53* in combination with genetic alterations of m^6^A regulatory genes to exhibit inferior EFS compared to patients without genetic alterations of these genes (Additional file 9: Figure S7a).

Because mutations, deletions, amplifications, and/or CNVs of m^6^A regulatory genes were relatively confined to patients with wild-type *FLT3* and *NPM1* (95.6%, Table 1), we determined whether these genetic alterations impact OS and EFS stratified by *FLT3* or *NPM1* mutation status. Inferior OS and EFS were observed in patients with wild-type *FLT3* who had ≥1 genetic alteration(s) of m^6^A regulatory gene(s) (*P* < 0.0001, Additional file 9: Figure S7b). Notably, these patients also had worse OS (*P* < 0.041) and EFS (*P* < 0.042) compared to patients who had mutant *FLT3* but no genetic alteration of m^6^A regulatory genes (Additional file 9: Figure S7b). Genetic alterations of m^6^A regulatory genes as a group were also significantly associated with a worse OS and EFS in patients with wild-type *NPM1* (*P* < 0.0001, Additional file 9: Figure S7c). Integration of molecular analyses of m^6^A regulatory genes may be useful to determine a poorer outcome in AML patients who have neither been classified as “poor risk” due to the presence of *FLT3* mutations [6, 7] nor better outcome conferred by *NPM1* mutations [8], particularly within a group of *TP53* wild-type patients.

In a multivariate Cox proportional hazard model that includes variables associated with poorer survival, genetic alterations of m^6^A regulatory genes as a group were not an independent prognostic factor for OS (Fig. 1d). However, genetic alterations of m^6^A regulatory genes did independently predict poorer OS (hazard ratio = 2.073; 95% CI, 1.13–3.80; *P* = 0.018) when *TP53* mutation was excluded from the model (Fig. 1d). Similar results were observed in multivariate analyses to predict EFS (Fig. 1d). Our results support a strong association between genetic alterations of m^6^A regulatory genes and *TP53* mutation. The fact that one is confounding the other in predicting patients’ outcome suggests that both may be complementary in the pathogenesis and/or maintenance of AML.

Identification of novel biomarkers and molecular targets to guide the development of anti-leukemic therapies remains a major challenge. Particularly for AML, the molecular markers to define subtypes and prognosis are under continuous refinement [7, 9]. Given that m^6^A modification to RNA has broad physiological functions, its impairment may be associated with the development and progression of diverse cancers, including leukemia. The current WHO classification highlights epigenetic modifiers as being mutated early during the clonal evolution of AML [9]. Novel genetic subgroups now include mutation in genes that encode splicing regulators, TP53, and other epigenetic modifiers [9].

Our present study is the first to determine the clinicopathological associations and impact of genetic alterations affecting m^6^A regulatory genes in AML. We found a striking association between genetic alterations of these genes as a group and *TP53* mutations (Table 1). Importantly, genetic alterations of m^6^A regulatory genes are associated with inferior outcome in AML patients, although this may be confounded by the adverse impact of *TP53* mutations on survival [10] (Additional files 6: Figure S4 and 9: Figure S7). It has been established that loss of the m^6^A methyltransferase, *METTL3*, resulted in alternative splicing and gene expression changes of >20 genes involved in the TP53 signaling pathway including *MDM2*, *MDM4*, and *P21* in a human liver cancer cell line [2]. It is plausible that genetic alterations of m^6^A modifiers, TP53, and/or its regulator/downstream targets contribute in complementary pathways to the pathogenesis and/or maintenance of AML. Further studies in larger AML cohorts would assist in confirming our findings and spur future research into the functional role of m^6^A RNA modification in AML and its link to tumorigenesis pathways, especially TP53 signaling.

## Additional Files

Additional file 1: Figure S1. Point mutation, deep deletion, amplification, shallow deletion, and copy number gain of m6A regulatory genes in hematological malignancies. (a) Percentage of leukemia samples with alteration to the genes encoding m6A regulators based on the Cancer Genome Atlas Research Network (TCGA) data. (b) Frequency of copy number gain or loss of the m6A regulatory genes in the TCGA AML samples. AML, Acute Myeloid Leukemia; MM, Multiple Myeloma; ALL, Acute Lymphoblastic Leukemia; CLL, Chronic Lymphocytic Leukemia. (PDF 361 kb)
Additional file 2: Table S1. AML samples with a mutation, deep deletion, amplification, copy number gain, and/or copy number loss of one or more genes encoding m^6^A regulatory enzymes. *Examples of potentially synergistic changes that may increase RNA m^6^A levels. (DOCX 103 kb)
Additional file 3: Figure S2. Associations between shallow deletion and copy number gain of m^6^A regulatory genes and their mRNA expression in the TCGA AML cohort. Relative mRNA expression is displayed as *Z*-score, which indicates the number of standard deviation away from the mean expression of the reference population represented by non-mutated diploid samples. Mann-Whitney *U* test was used to determine significance. (PDF 395 kb)
Additional file 4: Table S2. Clinical and molecular characteristics of TCGA AML patients with a deletion or copy number loss of the gene encoding an m^6^A eraser, *ALKBH5*. (DOCX 92 kb)
Additional file 5: Figure S3. Kaplan-Meier curves for overall and event-free survival of the TCGA AML patients by (a) age, (b) white blood cell (WBC) count at diagnosis, and (c) cytogenetic risk status. Log-rank test was used to determine significance. +, censored data. (PDF 482 kb)
Additional file 6: Figure S4. Kaplan-Meier curves for overall and event-free survival of the TCGA AML patients by (a) *DNMT3A* mutation status and (b) *TP53* mutation status. Log-rank test was used to determine significance. +, censored data. (PDF 424 kb)
Additional file 7: Figure S5. Kaplan-Meier curves for overall and event-free survival of patients with and without mutation and/or copy number variation (CNV) of m6A regulatory genes by (a) age >60 years and (b) age <60 years. Log-rank test was used to determine significance. +, censored data. (PDF 405 kb)
Additional file 8: Figure S6. Kaplan-Meier curves for overall and event-free survival of patients with and without mutation and/or copy number variation (CNV) of m6A regulatory genes by (A) unfavorable cytogenetic risk group, (B) white blood cell count (WBC) > median at diagnosis, and (C) mutated *DNMT3A*. Log-rank test was used to determine significance. +, censored data. (PDF 442 kb)
Additional file 9: Figure S7. Kaplan-Meier curves for overall and event-free survival of patients stratified by the status of m6A regulatory gene alterations in addition to (a) *TP53*, (b) *FLT3*, and (c) *NPM1* mutation status. Log-rank test was used to determine significance. WT, wild-type. +, censored data. (PDF 501 kb)
Additional file 10: Supplementary methods. (DOCX 79 kb)

## Abbreviations

ALL: Acute lymphoblastic leukemia
AML: Acute myeloid leukemia
CLL: Chronic lymphocytic leukemia
CNVs: Copy number variations
EFS: Event-free survival
m^6^A: Methylation of N^6^ adenosine
MM: Multiple myeloma
OS: Overall survival
TCGA: The Cancer Genome Atlas Research Network
