# Erratum to: Genetic alterations of m^6^A regulators predict poorer survival in acute myeloid leukemia

**DOI:** 10.1186/s13045-017-0419-x

**Published:** 2017-02-17

**Authors:** Chau-To Kwok, Amy D. Marshall, John E. J. Rasko, Justin J. L. Wong

**Affiliations:** 1 Gene & Stem Cell Therapy Program, Centenary Institute, University of Sydney, Camperdown, 2050 Australia; 2 Gene Regulation in Cancer Laboratory, Centenary Institute, University of Sydney, Camperdown, 2050 Australia; 3 Sydney Medical School, University of Sydney, Camperdown, NSW 2006 Australia; 4 Cell and Molecular Therapies, Royal Prince Alfred Hospital, Camperdown, 2050 Australia

## Erratum

The original article [1] contained an error whereby the formatting of Table 1 was not presented correctly; this error has now been corrected.

**Table 1:** Clinical and molecular characteristics of TCGA AML patients according to the mutation and/or copy number variation status of genes encoding m^6^A regulatory enzymes

	Mutation and/or CNV			CNV only^a^			Mutation		
	Yes (*n* = 23)	No (*n* = 168)	*P*	Yes (*n* = 18)	No (*n* = 168)	*P*	Yes (*n* = 5)	No (*n* = 186)	*P*
Age			0.083			0.193			0.205
Median (range)	65 (18–81)	57 (21–88)		62.5 (18–81)	57 (21–88)		65 (45–76)	57.5 (18–88)	
Sex, no. (%)			0.123			0.321			0.376
Male	16 (8.4)	87 (45.5)		12 (6.5)	87 (46.8)		4 (2.1)	99 (51.8)	
Female	7 (3.7)	81 (42.4)		6 (3.2)	81 (43.5)		1 (0.5)	87 (45.5)	
BM blast			0.072			**0.038**			0.915
Median % (range)	60 (30–97)	73 (30–100)		54 (30–97)	73 (30–100)		75 (33–90)	72 (30–100)	
WBC, ×10^3^/mm^3^			0.084			**0.047**			0.889
Median (range)	5.4 (0.7–202.7)	17.5 (0.4–298.4)		5.2 (2.3–101.3)	17.45 (0.4–298.4)		14.5 (2.3–101.3)	15.6 (0.4–298.4)	
Cytogenetic risk, no. (%)			**<0.0001**			**<0.0001**			0.483
Favorable	0 (0)	37 (19.4)		0 (0)	37 (19.9)		0 (0)	37 (19.4)	
Intermediate	4 (2.1)	105 (55)		1 (0.5)	105 (56.5)		3 (1.6)	106 (55.5)	
Unfavorable	19 (9.9)	21 (11)		17 (9.1)	21 (11.3)		2 (1)	38 (19.9)	
Missing data	0 (0)	5 (2.6)		0 (0)	5 (2.6)		0 (0)	5 (2.6)	
Mutation, no./total no. (%)									
* FLT3*	1/23 (4.3)	53/168 (31.5)	**0.005**	0/18 (0)	53/168 (31.5)	**0.002**	1/5 (20)	53/186 (28.4)	1.000
* NPM1*	1/23 (4.3)	51/168 (30)	**0.006**	0/18 (0)	51/168 (30.3)	**0.004**	1/5 (20)	51/186 (27.4)	1.000
* DNMT3A*	4/23 (17.4)	43/168 (25.6)	0.453	2/18 (11.1)	43/168 (25.6)	0.249	2/5 (40)	45/186 (24.2)	0.598
* IDH1* or *IDH2*	1/23 (4.3)	34/168 (20.2)	0.084	0/18 (0)	34/168 (20.2)	**0.048**	1/5 (20)	34/186 (18.3)	1.000
* NRAS* or *KRAS*	3/23 (13)	20/168 (11.9)	0.744	3/18 (16.7)	20/168 (11.9)	0.471	0/5 (0)	23/186 (12.4)	1.000
* RUNX1*	2/23 (8.7)	17/168 (10.1)	1.000	0/18 (0)	17/168 (10.1)	0.380	2/5 (40)	17/186 (9.1)	0.078
* TET2*	1/23 (4.3)	15/168 (8.9)	0.698	1/18(5.6)	15/168 (8.9)	1.000	0/5 (0)	16/186 (8.6)	1.000
* TP53*	15/23 (65.2)	1/168 (0.6)	**<0.0001**	13/18 (72.2)	1/168 (0.6)	**<0.0001**	2/5 (40)	14/186 (7.5)	0.057
* CEBPA*	2/23 (8.7)	10/168 (6.0)	0.641	2/18 (11.1)	10/168 (6.0)	0.327	0/5 (0)	12/186 (6.5)	1.000
* WT1*	0/23 (0)	12/168 (7.1)	0.366	0/18 (0)	12/168 (7.1)	0.610	0/5 (0)	12/186 (6.5)	1.000
* PTPN11*	2/23 (8.7)	6/168 (3.6)	0.248	2/18 (11.1)	6/168 (3.6)	0.175	0/5 (0)	8/186 (4.3)	1.000
* KIT*	1/23 (4.3)	6/168 (3.6)	0.599	0/18 (20)	6/168 (3.6)	1.000	1/5 (20)	6/186 (3.2)	0.172

Significant *P* values are in bold

*CNV* copy number variation, *BM* bone marrow, *WBC* white blood cell

^a^Excluding samples with m^6^A regulatory gene mutations

Furthermore, the error was mistakenly carried forward by the production team handling this article, and thus was not the fault of the authors.

## References

[1] Kwok (2017). Genetic alterations of m^6^A regulators predict poorer survival in acute myeloid leukemia. J Hematol Oncol.

