# Supplementary figures and images for: Genetic alterations of m6A regulators predict poorer survival in acute myeloid leukemia
Source: J Hematol Oncol. 2017 Feb 2;10:39. doi: 10.1186/s13045-017-0410-6 (PMC5290707; doi:10.1186/s13045-017-0410-6)

Fig. S1

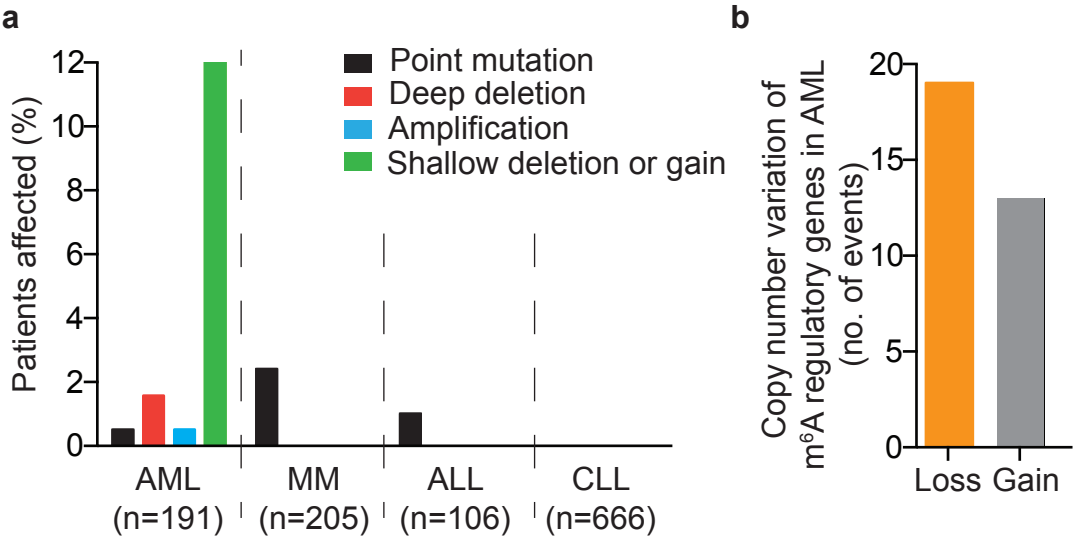

Supplement: Additional file 1: Figure S1. — Point mutation, deep deletion, amplification, shallow deletion, and copy number gain of m6A regulatory genes in hematological malignancies. (a) Percentage of leukemia samples with alteration to the genes encoding m6A regulators based on the Cancer Genome Atlas Research Network (TCGA) data. (b) Frequency of copy number gain or loss of the m6A regulatory genes in the TCGA AML samples. AML, Acute Myeloid Leukemia; MM, Multiple Myeloma; ALL, Acute Lymphoblastic Leukemia; CLL, Chronic Lymphocytic Leukemia. (PDF 361 kb) [file 13045_2017_410_MOESM1_ESM.pdf]

**Fig. S2**

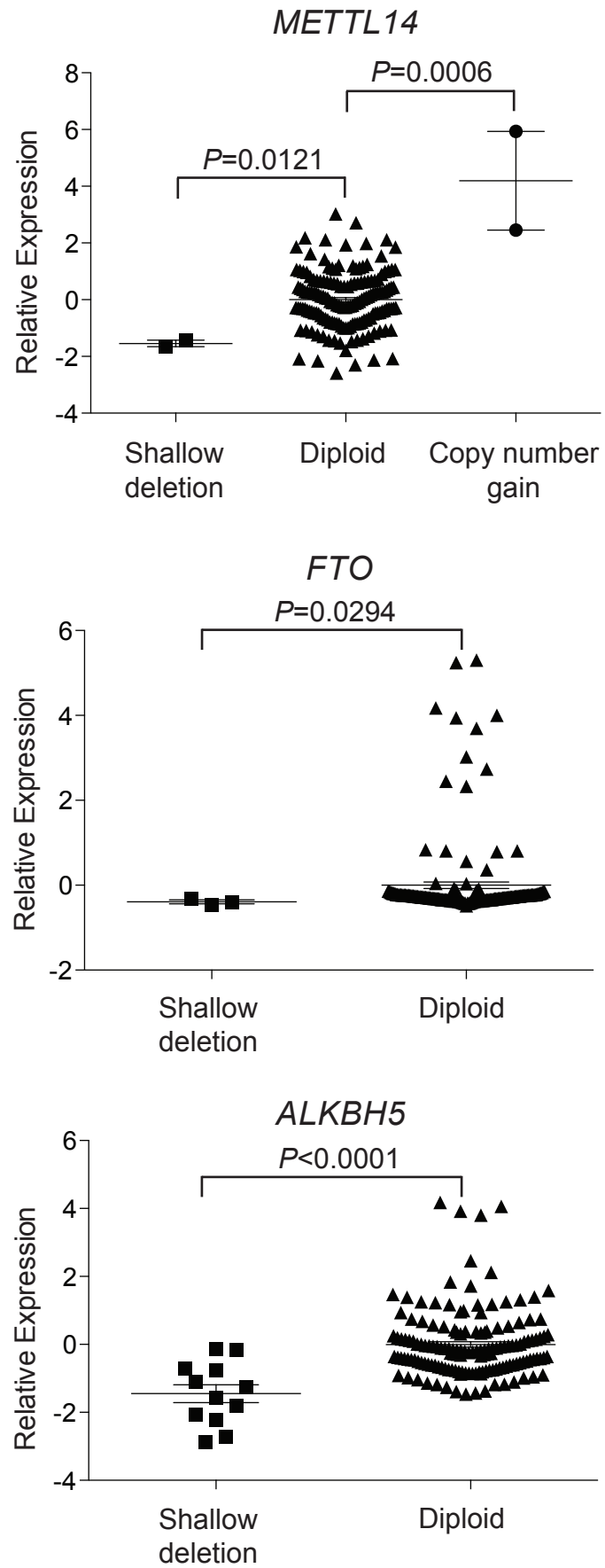

Supplement: Additional file 3: Figure S2. — Associations between shallow deletion and copy number gain of m6A regulatory genes and their mRNA expression in the TCGA AML cohort. Relative mRNA expression is displayed as Z-score, which indicates the number of standard deviation away from the mean expression of the reference population represented by non-mutated diploid samples. Mann-Whitney U test was used to determine significance. (PDF 395 kb) [file 13045_2017_410_MOESM3_ESM.pdf]

**Fig. S3**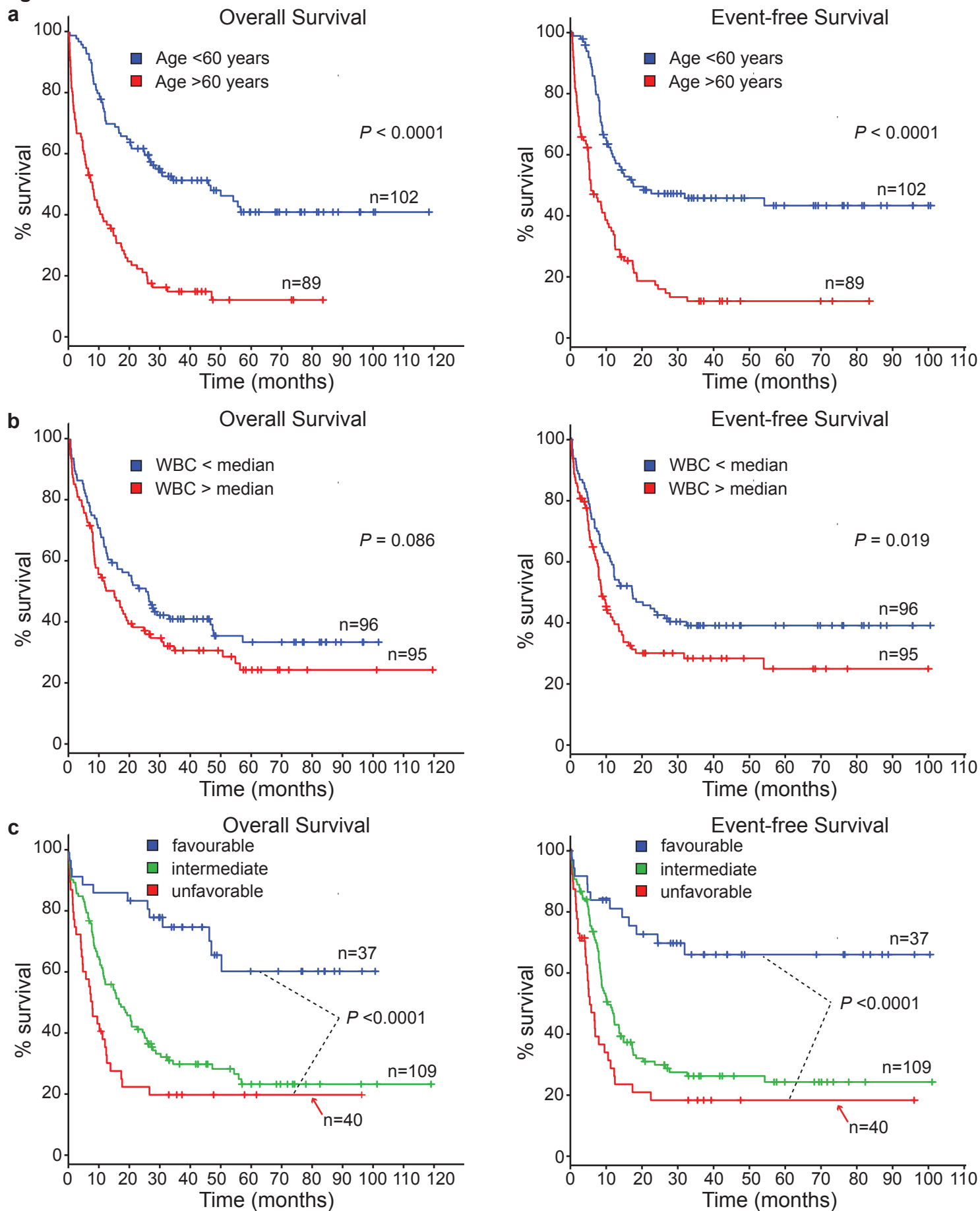

Supplement: Additional file 5: Figure S3. — Kaplan-Meier curves for overall and event-free survival of the TCGA AML patients by (a) age, (b) white blood cell (WBC) count at diagnosis, and (c) cytogenetic risk status. Log-rank test was used to determine significance. +, censored data. (PDF 482 kb) [file 13045_2017_410_MOESM5_ESM.pdf]

**Fig. S4**

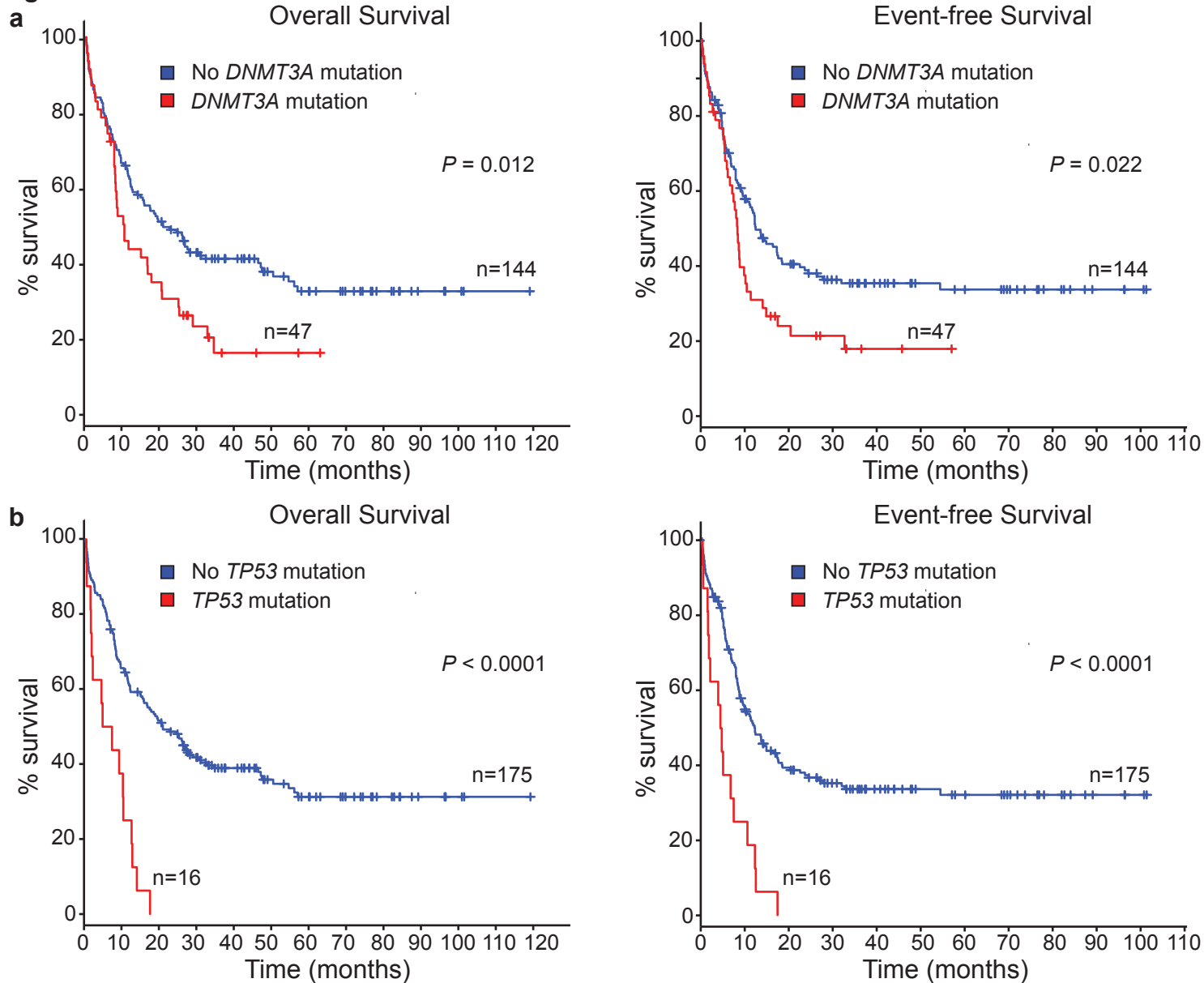

Supplement: Additional file 6: Figure S4. — Kaplan-Meier curves for overall and event-free survival of the TCGA AML patients by (a) DNMT3A mutation status and (b) TP53 mutation status. Log-rank test was used to determine significance. +, censored data. (PDF 424 kb) [file 13045_2017_410_MOESM6_ESM.pdf]

**Fig. S5**

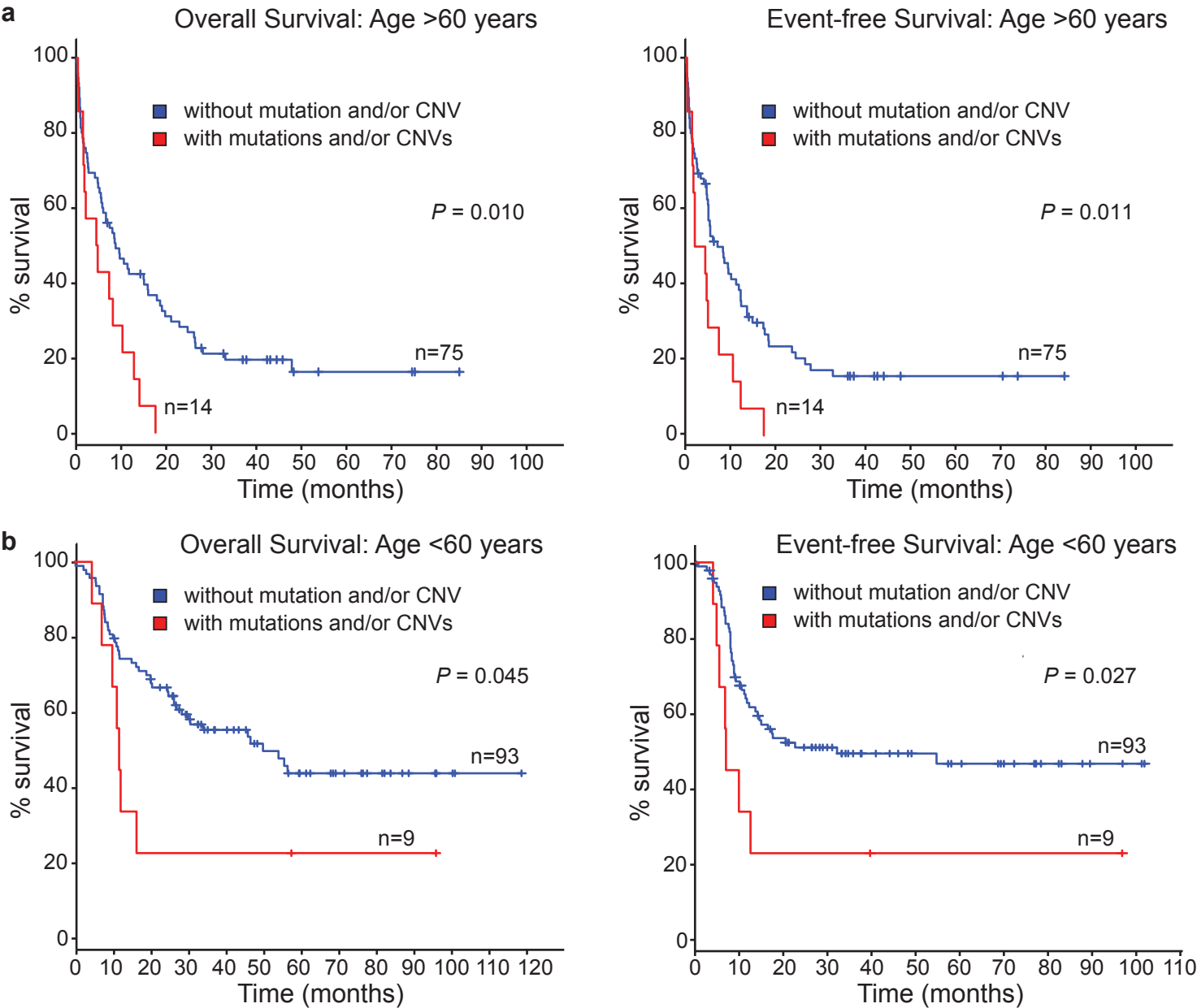

Supplement: Additional file 7: Figure S5. — Kaplan-Meier curves for overall and event-free survival of patients with and without mutation and/or copy number variation (CNV) of m6A regulatory genes by (a) age >60 years and (b) age <60 years. Log-rank test was used to determine significance. +, censored data. (PDF 405 kb) [file 13045_2017_410_MOESM7_ESM.pdf]

**Fig. S6**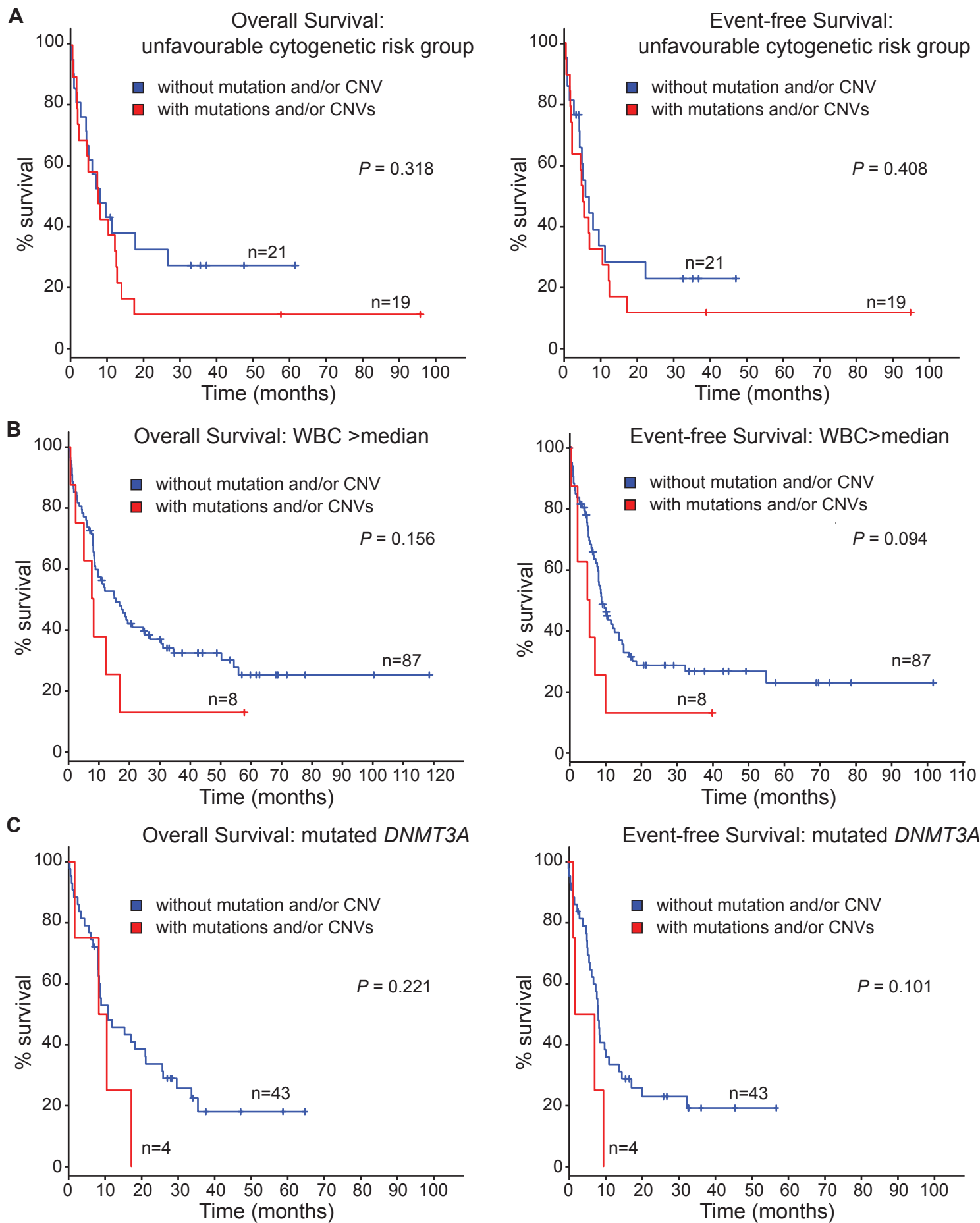

Supplement: Additional file 8: Figure S6. — Kaplan-Meier curves for overall and event-free survival of patients with and without mutation and/or copy number variation (CNV) of m6A regulatory genes by (A) unfavorable cytogenetic risk group, (B) white blood cell count (WBC) > median at diagnosis, and (C) mutated DNMT3A. Log-rank test was used to determine significance. +, censored data. (PDF 442 kb) [file 13045_2017_410_MOESM8_ESM.pdf]

**Fig. S7**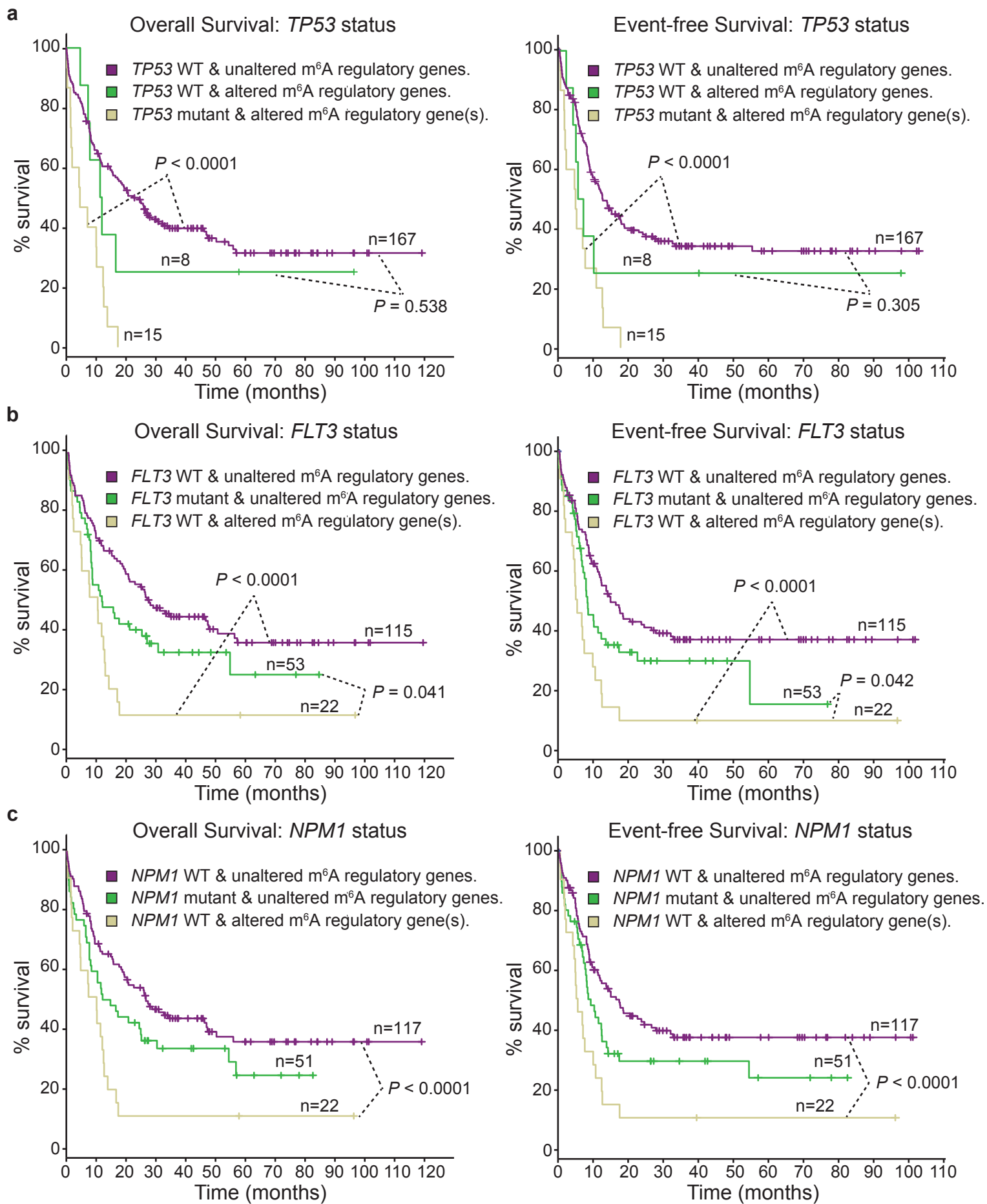

Supplement: Additional file 9: Figure S7. — Kaplan-Meier curves for overall and event-free survival of patients stratified by the status of m6A regulatory gene alterations in addition to (a) TP53, (b) FLT3, and (c) NPM1 mutation status. Log-rank test was used to determine significance. WT, wild-type. +, censored data. (PDF 501 kb) [file 13045_2017_410_MOESM9_ESM.pdf]
