# Supplementary material for: Genetic alterations of m6A regulators predict poorer survival in acute myeloid leukemia
Source: J Hematol Oncol. 2017 Feb 2;10:39. doi: 10.1186/s13045-017-0410-6 (PMC5290707; doi:10.1186/s13045-017-0410-6)
Supplement: Additional file 2: Table S1. — AML samples with a mutation, deep deletion, amplification, copy number gain, and/or copy number loss of one or more genes encoding m6A regulatory enzymes. *Examples of potentially synergistic changes that may increase RNA m6A levels. (DOCX 103 kb) [file 13045_2017_410_MOESM2_ESM.docx]

|  | m^6^A writer | | m^6^A eraser | | m^6^A reader | |
| --- | --- | --- | --- | --- | --- | --- |
| TCGA-ID | *METTL3* | *METTL14* | *FTO* | *ALKBH5* | *YTHDF1* | *YTHDF2* |
| 2983 |  |  |  |  |  |  |
| 2857 |  |  |  |  |  |  |
| 2820* |  |  |  |  |  |  |
| 2952 |  |  |  |  |  |  |
| 2813 |  |  |  |  |  |  |
| 2941 |  |  |  |  |  |  |
| 2945* |  |  |  |  |  |  |
| 2883 |  |  |  |  |  |  |
| 2849* |  |  |  |  |  |  |
| 2855* |  |  |  |  |  |  |
| 2878 |  |  |  |  |  |  |
| 2933 |  |  |  |  |  |  |
| 2908 |  |  |  |  |  |  |
| 2860 |  |  |  |  |  |  |
| 2935 |  |  |  |  |  |  |
| 2920 |  |  |  |  |  |  |
| 2955 |  |  |  |  |  |  |
| 2838 |  |  |  |  |  |  |
| 2917 |  |  |  |  |  |  |
| 2868 |  |  |  |  |  |  |
| 2938 |  |  |  |  |  |  |
| 2904 |  |  |  |  |  |  |
| 2943 |  |  |  |  |  |  |

**Table S1.** AML samples with a point mutation, deep deletion, amplification, shallow deletion and/or copy number gain of one or more genes encoding m^6^A regulatory enzymes. * Examples of potentially synergistic changes that may increase RNA m^6^A levels.

Shallow deletion

Point mutation

|  |
| --- |

Copy number gain

Deep deletion

|  |
| --- |

Amplification

|  |
| --- |
