# Supplementary material for: Genetic alterations of m6A regulators predict poorer survival in acute myeloid leukemia
Source: J Hematol Oncol. 2017 Feb 2;10:39. doi: 10.1186/s13045-017-0410-6 (PMC5290707; doi:10.1186/s13045-017-0410-6)
Supplement: Additional file 4: Table S2. — Clinical and molecular characteristics of TCGA AML patients with a deletion or copy number loss of the gene encoding an m6A eraser, ALKBH5. (DOCX 92 kb) [file 13045_2017_410_MOESM4_ESM.docx]

**Table S2.** Clinical and molecular characteristics of TCGA AML patients with a deletion or copy number loss of the gene encoding an m^6^A eraser, *ALKBH5*.

|  | Deletion or copy number loss of *ALKBH5* | | |  |
| --- | --- | --- | --- | --- |
|  | Yes (n=14) | No (n=177) | *P* | |
| Age |  |  | 0.477 | |
| Median (range) | 59 (18-81) | 58 (21-88) |  | |
| Sex, no.(%) |  |  | 0.092 | |
| Male | 11 (5.8) | 92 (48.2) |  | |
| Female | 3 (1.6) | 85 (44.5) |  | |
| AML-FAB subtype, no. (%) |  |  |  | |
| M0 | 3 (1.6) | 16 (8.4) |  | |
| M1 | 1 (0.5) | 41 (21.5) |  | |
| M2 | 4 (2.1) | 39 (20.4) |  | |
| M3 | 0 (0) | 20 (10.5) |  | |
| M4 | 3 (1.6) | 36 (18.8) |  | |
| M5 | 0 (0) | 21 (11) |  | |
| M6 | 1 (0.5) | 1 (0.5) |  | |
| M7 | 1 (0.5) | 2 (1) |  | |
| Other | 1 (0.5) | 1 (0.5) |  | |
| BM blast |  |  | 0.130 | |
| Median % (range) | 59 (30-90) | 73 (30-100) |  | |
| WBC, ×10^3^ per mm^3^ |  |  | 0.686 | |
| Median (range) | 12.2 (0.8-202.7) | 16 (0.4-298.4) |  | |
| Cytogenetic risk, no. (%) |  |  | **<0.0001** | |
| Favourable | 0 (0) | 37 (19.4) |  | |
| Intermediate | 2 (1.0) | 107 (56) |  | |
| Unfavourable | 12 (6.3) | 28 (14.7) |  | |
| Missing data | 0 (0) | 5 (2.6) |  | |
| Mutation, no./total no. (%) |  |  |  | |
| *FLT3* | 0/14 (0) | 54/177 (30.5) | **0.012** | |
| *NPM1* | 0/14 (0) | 52/177 (29.4) | **0.013** | |
| *DNMT3A* | 2/14 (14.3) | 45/177 (25.4) | 0.524 | |
| *IDH1* or *IDH2* | 0/14 (0) | 35/177 (19.8) | 0.077 | |
| *NRAS* or *KRAS* | 2/14 (14.3) | 21/177 (11.9) | 0.678 | |
| *RUNX1* | 1/14 (7.1) | 18/177 (10.2) | 1.000 | |
| *TET2* | 1/14 (7.1) | 15/177 (8.5) | 1.000 | |
| *TP53* | 8/14 (57.1) | 8/177 (4.5) | **<0.0001** | |
| *CEBPA* | 1/14 (7.1) | 11/177 (6.2) | 1.000 | |
| *WT1* | 0/14 (0) | 12/177 (6.8) | 0.605 | |
| *PTPN11* | 1/14 (7.1) | 7/177 (4) | 0.463 | |
| *KIT* | 0/14 (0) | 7/177 (4) | 1.000 | |

BM, bone marrow; WBC, white blood cell; M0, AML without differentiation; M1, AML with minimal differentiation; M2, AML with maturation; M3, acute promyelocytic leukemia, M4, acute myelomonocytic leukemia; M5, acute monoblastic and monocytic leukemia; M6/M7, acute erythroid leukemia/acute megakaryoblastic leukemia. Significant *P* values are in bold.
