# Supplementary material for: Genetic alterations of m6A regulators predict poorer survival in acute myeloid leukemia
Source: J Hematol Oncol. 2017 Feb 2;10:39. doi: 10.1186/s13045-017-0410-6 (PMC5290707; doi:10.1186/s13045-017-0410-6)
Supplement: Additional file 10: — Supplementary methods. (DOCX 79 kb) [file 13045_2017_410_MOESM10_ESM.docx]

**Supplementary Methods**

**Patient data**

The clinicopathological, mutation, deletion, amplification, copy number variation and/or survival data from leukemia patients are available via the cBioportal, the TCGA data portal and/or reported in a previous publication. Of the 200 AML patients in the TCGA cohort, matched mutation, deletion, amplification and copy number variation data are available for 191 patients via cBioportal. We therefore included only these patients in our analyses.

**Statistical analyses**

Categorical variables were compared using the chi-square test or Fisher’s exact test. Fisher-Freeman-Halton test was performed for contingency tables that are larger than 2x2. The Mann-Whitney *U* test was used for the comparison of continuous variables that were not normally distributed. The Kaplan-Meier analysis and the log-rank test were used to estimate the distribution of overall (OS) and event-free survival (EFS) and to compare differences between survival curves, respectively. Multivariate analyses were performed using the Cox proportional hazards models. Variables with *P*<0.05 (log-rank test) in univariate analysis for OS and/or EFS were included in the models. All statistical analyses were performed using the SPSS statistical package, version 24.0 (IBM, Corp.). For all tests, differences were considered significant at *P*<0.05 (two-sided).
